# The Dark Pigment in the Sesame (*Sesamum indicum* L.) Seed Coat: Isolation, Characterization, and Its Potential Precursors

**DOI:** 10.3389/fnut.2022.858673

**Published:** 2022-02-28

**Authors:** Senouwa Segla Koffi Dossou, Zishu Luo, Zhijian Wang, Wangyi Zhou, Rong Zhou, Yanxin Zhang, Donghua Li, Aili Liu, Komivi Dossa, Jun You, Linhai Wang

**Affiliations:** ^1^Key Laboratory of Biology and Genetic Improvement of Oil Crops of the Ministry of Agriculture, Oil Crops Research Institute of the Chinese Academy of Agricultural Sciences, Wuhan, China; ^2^Laboratoire de Physiologie et de Biotechnologie Végétales, Faculté Des Sciences, Université de Lomé, Lomé, Togo; ^3^CIRAD, UMR AGAP Institut, Montpellier, France; ^4^UMR AGAP Institut, Univ Montpellier, CIRAD, INRAE, Institut Agro, Montpellier, France

**Keywords:** sesame, melanin, seed coat color, metabolite profiling, LC-MS/MS

## Abstract

Sesame is a worldwide oilseed crop used in the food pharmacy. Its seed phenotypes determine the seed quality values. However, a thorough assessment of seed coat metabolites is lacking, and the dark pigment in the seed coat is not well-characterized. Herein, we report the isolation of melanin by the alkali method from the black and brown sesame seeds. Physicochemical methods, including scanning electron microscopy (SEM), solubility, precipitation, UV-Vis spectroscopy, Fourier transform infrared (FT-IR) spectroscopy, and thermogravimetric-differential scanning calorimetry (TG-DSC), were used to characterize the sesame melanins. The results clearly showed that the isolated pigments were similar to melanin from other sources. Both melanins were heat-stable and exhibited numerous characteristic absorption peaks. Through a comprehensible LC-MS/MS-based metabolome profiles analysis of NaOH and methanol extracts of black and white sesame seeds, caffeic, protocatechuic, indole-carboxylic, homogentisic, ferulic, vanillic, and benzoic acids were identified as the potential precursors of the sesame melanin. Our findings widen our understanding of dark seeds pigmentation in sesame. Furthermore, they show that black sesame seeds are promising sources of edible melanin for food and biotechnological applications.

## Introduction

Food and nutrition play critical roles in the management, prevention, treatment, and in some cases, reversal of human diseases (1, 2). Accordingly, several advanced analytical methods have been developed to access food's quality and implement the food pharmacy, which in turn may increase consumption of healthful diets (3). Among them, metabolomics has emerged as the most important tool for chemical characterization of the metabolome underpinning the phenotype of organisms through the qualitative and quantitative analysis of all metabolites and the evaluation of changes in the metabolite profiles due to stresses or perturbations (4). It provides high-quality data on food biochemistry and helps understand biological processes (5). Metabolomics experiments usually consist of four steps: samples collection, extraction, analysis of the extract, data reduction, and statistical analyses. Of them, the extraction is the critical step that greatly affects the results (6). Only a comprehensive and repeatable extraction method may provide reliable data since the metabolites that will be identified are those that were extracted, and the conclusions will be made based on incomplete information (6, 7). In sesame, metabolomics analysis of methanol extracts identified a wide range of metabolites, including nutrients and diverse bioactive compounds, and helped understand some processes such as seed antioxidant activities and the pigmentation of flowers (8–10). However, the same analytical method applied to different colored sesame seeds unrevealed the pigments responsible for seeds' coat color variation (10). Thus, the need to evaluate other solvents and identify the most efficient for metabolites profiling analysis of sesame seeds.

Seed coat color is an important agronomic trait in sesame that determines the seed's biochemistry and influences its nutritional and therapeutical values (8, 11). It defines the consumers' preferences and negatively impacts sesame breeders' effort to meet seeds' market demands. Among the different colored sesame seeds, the dark ones are the most preferred. Thus, it is of great importance to identify the dark pigment in the sesame seeds for a better understanding of the sesame seed coat pigmentation. The black and brown colors of seeds are usually attributed to proanthocyanidins and/or melanins (12–14). In sesame, reports suggested that the dark pigment might be melanin (8, 15–19). Melanins are structurally complex polymeric pigments produced in all biological systems, including animals, plants, and microorganisms (20–22). They display different colors, including black, brown, to yellow, and are characterized as heterogenic polymers biosynthesized from oxidative polymerization of phenolic and hydroxyindole derivatives (20, 21). Natural melanins are hydrophobic and negatively charged macromolecules that are dissolvable in alkaline solutions and insoluble in water, acids, or organic solvents (23, 24). Animals and microorganisms' melanins biosynthesis, characteristics, and biological functions have been well-studied (20, 23). In contrast, limited studies were focused on melanin sources in plants. Melanin has been identified as the dark pigment in black oat (14), watermelon seeds (25), persimmon fruit skin (24), black garlic (26), and was associated with the seed coat cracking phenotypes in peanuts (27). These studies revealed that polyphenol oxidase (PPO), a nearly ubiquitous oxidoreductase enzyme in Angiosperms, is essential for melanins biosynthesis in plants (24, 25, 27, 28). It catalyzes the oxidation of phenolics to quinones, leading to enzymatic browning. PPO was identified as the key candidate causative gene of black seed pigmentation in sesame (13, 16, 18). Although the key biosynthesis gene of melanin in plants is identified, the biosynthetic pathway is still unclear. According to some studies, the potential precursors of melanin in plants are catechol, ferulic acid, gallic acid, caffeic acid, protocatechuic acid, homogentisic acid, and epigallocatechin gallate (14, 24, 26).

The typical protocol of melanin isolation includes their alkaline extraction followed by precipitation in acid conditions (14, 24, 26). To confirm the melanic nature of isolated pigments, some chemical and physical tests, including solubility, oxidation, precipitation, spectroscopy (UV-Vis and Fourier transform infrared, FT-IR), and thermal stability, are broadly used (13). In this study, the dark pigment of black and brown sesame seeds was isolated and characterized. Moreover, through a comprehensive metabolome profile analysis of black and white sesame seeds, we identified the potential precursors of the sesame melanin. This work represents a new guideline for further research on the sesame seed's coat pigmentation. Furthermore, it provides valuable data for future studies toward the identification of the most efficient extraction solvent for metabolomics analysis in sesame.

## Materials and Methods

### Plant Materials

The sesame varieties (Supplementary Figure S1) used in this study were provided by the National Sesame Medium-term Genebank (Wuhan, China). They were cultivated from June to October 2020 under identical growth conditions at the experimental station of the Oil Crops Research Institute of the Chinese Academy of Agricultural Sciences (OCRI-CAAS) located in Wuhan, China (N 30.57°, E 114.30°, 27 m altitude). Seed samples were prepared after harvest by mixing seeds from 15 individual plants. Samples used for the melanin extraction were kept at the seeds room of the Institute, and those for the widely targeted metabolomic profiling analysis were stored at −80°C.

### Melanin Isolation

The melanin was extracted from the black and brown seeds following previously reported methods with some little modifications (23, 24, 27). Briefly, 1 g of ground seeds was solubilized in 10 ml of 0.5 mol/L NaOH for 4 h, which generated a dark brown solution. After centrifugation at 5,000 rpm for 5 min, the supernatant was collected and filtered. The extraction process was repeated twice, and the supernatants were combined. The pooled supernatant was centrifuged again before the seed melanin was precipitated from the aqueous solution by acidification with 6 mol/L HCl at pH 2. The mixture was incubated for at least 12 h at room temperature. The crude melanin was obtained after centrifugation at 10,000 rpm for 10 min. The precipitate was redissolved in 0.5 mol/L NaOH, and 6 mol/L HCl was added again to ensure a higher purity of the melanin. Then, the precipitate of the seed melanin was washed thoroughly with distilled water until the supernatant pH became neutral. Thereafter the precipitate was sub sequentially washed with chloroform and ethanol to discard the residues of oil and other seed components. Finally, the crude seed melanin was dried overnight at 55°C, weighted, and kept for further analysis.

### Melanin Characterization

The methods used to characterize the black and brown sesame seed's melanin included the morphology and size distribution, solubility, precipitation, UV–Vis absorption spectroscopy, Fourier transform infrared spectroscopy (FT-IR), and thermogravimetric-differential scanning calorimetry (TG-DSC).

The morphology and size distribution of the black and brown sesame seeds melanin pigments were examined using scanning electron microscopy (SEM, S-4800, Hitachi Co., Ltd., Matsuda, Japan) as per Wang et al. (26). Prior to the observation, the melanin powders were coated with a platinum (Pt) layer to increase the conductivity of the samples.

The seed melanin solubility, precipitation, and absorbance were analyzed following the method reported by Qi et al. (24) with little modification. The dried melanins were suspended in aqueous (distilled water and 0.5 M NaOH) and organic solvents (methanol, ethanol, chloroform, isopropyl alcohol, and hexane). The mixture of the melanin and solvent (10 mg/ml) in 10 mL tubes was kept in a water bath at 25°C for 4 h. The solubility was recorded after the mixture was centrifuged at 5,000 rpm for 5 min. For the precipitation, 125 μL of 10% FeCl_3_ and 80 μL of 6 mol/L HCl were added to 500 μL of the melanin solutions. The mixtures were kept at room temperature for 30 min followed by centrifugation at 10,000 rpm for 5 min. For the UV-Vis absorption spectroscopy, 10 mg of the seed and synthetic melanin was dissolved in 10 mL of 0.5 M NaOH. The UV-Vis absorption spectra of the solution were recorded on a UV- 2,450 spectrophotometer (Shimadzu, Japan) in the 220–900 nm range. The synthetic melanin (MelSYNTH, M8631) was purchased from Sigma-Aldrich (St. Louis, MO, USA). It is an L-Dopa melanin obtained from L-tyrosine in the presence of H_2_O_2_.

The FT-IR spectra of the sample were acquired using a Nicolet Is10 FT-IR spectrometer (Thermo Scientific, USA) equipped with a diamond ATR accessory as per Qi et al. (24). The measurements were conducted in the 4,000–600 cm^−1^ range with a 4 cm^−1^ optical resolution.

The TG-DSC curves were registered on NETZSCH STA 449 C (Netzsch-Gerätebau GmbH, Selb, Germany) thermal analysis instruments (29, 30). Each color seed melanin sample (10 mg) was heated from room temperature to 1,000°C at a heating rate of 10°C/min under a nitrogen flow.

### LC-MS-Based Widely Targeted Metabolomics Analysis of Different Solvent Extracts of Black and White Sesame Seeds

Freeze-dried white and black sesame seed samples (Supplementary Table S1 and Supplementary Figure S1) were grounded using a mixer mill (MM 400, Retsch, Haan, Germany) with a zirconia bead for 1.5 min at 30 Hz. Further, 100 mg of lyophilized powder of each sample was extracted overnight at 4°C in 1.2 ml of 0.5 M NaOH and 70 % methanol (Supplementary Table S1). Next, they were centrifugated at 12,000 rpm for 10 min, and the supernatants were collected separately and filtrated through a 0.22 μm micropore membrane (SCAA-104, ANPEL, Shanghai, China). The extracts were conserved at −20°C up to the ultra-high-performance liquid chromatography coupled with electrospray ionization tandem triple quadrupole mass spectrometry (UHPLC-ESI-QqQLIT-MS/MS) analysis (9, 31). The QC (quality control) samples were prepared by mixing the different seed extracts. The metabolomics analysis and the qualitative and quantitative analyses of metabolites were performed similarly as we reported recently (9, 10).

### Statistical Analysis

The metabolomics data quality was prior assessed, and substances with large deviations (CV value > 0.5) were eliminated. Unsupervised PCA (principal component analysis) and HCA (hierarchical cluster analysis) were performed in R (version 3.5.0) using the statistics function prcomp and the R package pheatmap (www.r-project.org). Normalized data by log2-transformation was used for Orthogonal Partial Least Squares-Discriminant Analysis (OPLS-DA) in R. The *p* and fold change (FC) values were set to 0.05 and 2.0, respectively. The differential metabolites were filtered out based on the FC and VIP ≥ 1. Venn diagrams were constructed to illustrate differences in metabolite numbers. Differential metabolites were annotated using the KEGG Compound database (http://www.kegg.jp/kegg/compound/) and further mapped to the KEGG Pathway database (http://www.kegg.jp/kegg/pathway.html). Pathways with significantly regulated metabolites were considered metabolite sets enrichment analysis (MSEA). The significance of the enrichment was determined by the hypergeometric test's *p*-values. Besides, GraphPad Prism v9.0.0121 (GraphPad Software Inc., La Jolla, CA, USA) was used for some graphing.

## Results

### Variation and Morphology of Black and Brown Sesame Seeds Melanin

**Figure 1:**
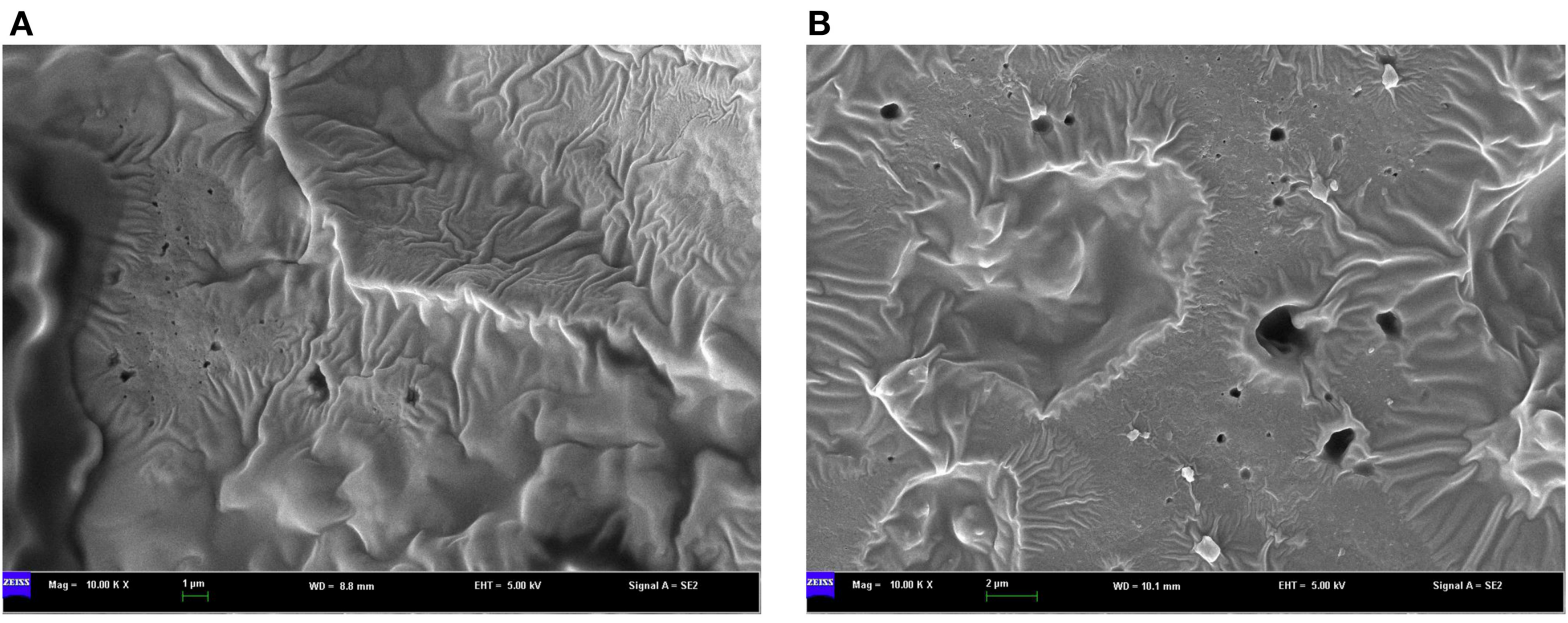
SEM micrographs of melanin isolated from **(A)** black sesame seeds and **(B)** brown sesame seeds.

**Figure 2:**
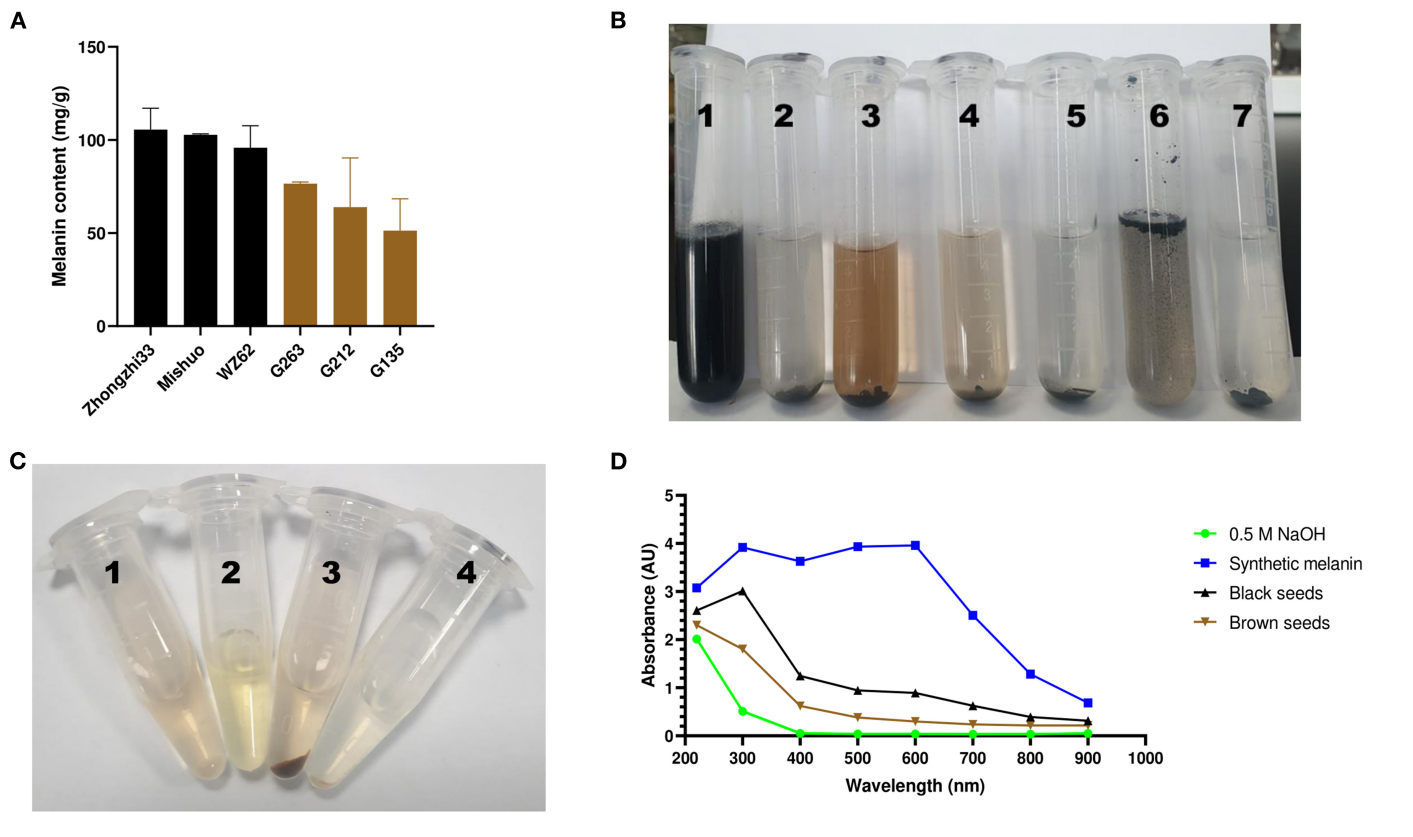
Variation, chemical, and UV-Vis absorbance characteristics of sesame melanins. **(A)** variation of melanin content in black and brown sesame seeds. **(B)** Solubility of the black sesame melanin. The serial numbers 1–7 on the tube correspond to melanin in an alkaline solution (0.5 M NaOH), H_2_O (pH 6.8), methanol (80%), ethanol, isopropyl alcohol, chloroform, and hexane, respectively. **(C)** Precipitation of the brown sesame seed melanin. The serial numbers 1–4 correspond to melanin in a sodium hydroxide solution (1 mg/mL) + 125 μL of ddH2O, 500 μL of ddH2O + 125 μL of 10 % FeCl_3_, melanin in a sodium hydroxide solution (1 mg/mL) + 125 μL of 10 % FeCl_3_, and melanin in a sodium hydroxide solution (1 mg/mL, pH 8.0) + 80 μL of 6 mol/L HCl. **(D)** UV–spectroscopy absorbance spectra of a synthetic melanin and the melanin pigments isolated from black and brown sesame seeds.

To determine whether the dark sesame seed pigment is melanin, we followed the previously described melanin isolation method and extracted melanin from the black and brown sesame seeds. Each colored seed was represented by three varieties (Supplementary Figure S1), and the experiment was conducted in triplicate. The morphology of melanins is an important parameter to compare melanin samples from various sources. The SEM images showed that the black and brown sesame melanin samples exhibit amorphous form without self-organization (Figures 1A,B). The melanin content in the black and brown sesame seeds ranged from 95.8 to 105.5 mg/g and 51.3 to 76.5 mg/g, respectively (Figure 2A).

### Chemical and UV-Vis Spectroscopic Analyses

We tested the extracted pigments' solubility, precipitation, and absorbance to confirm their melanin nature. The black sesame pigment showed a perfect solubility in an alkaline solution (0.5 M NaOH) and could not be dissolved in water, isopropyl alcohol, chloroform, and hexane (Figure 2B). However, it was slightly soluble in methanol (80%) and ethanol. The extracted pigments exhibited precipitation in 10% FeCl3 and 6 mol/L HCl solutions (Figure 2C). The extracted pigments and synthetic melanin were dissolved in 0.5 M NaOH (1 mg/ml) to obtain their absorption in different spectra from 220 to 900 nm. The absorbance spectrum of the black seed extract included a linear increase of the absorbance from 900 to 400 nm, an exponential increase from 400 to 300 nm, then a decrease from 300 to 220 nm (Figure 2D). The brown seed melanins' absorbance spectra comprised a linear increase of absorbance from 900 to 400 nm, followed by an exponential increase of absorbance from 400 to 220 nm. In contrast to the black seed extract, the synthetic melanin absorbance spectrum showed an exponential increase from 900 to 600 nm and was almost stable between 600 and 300 nm (Figure 2D).

### FT-IR and Thermal Stability

**Figure 3:**
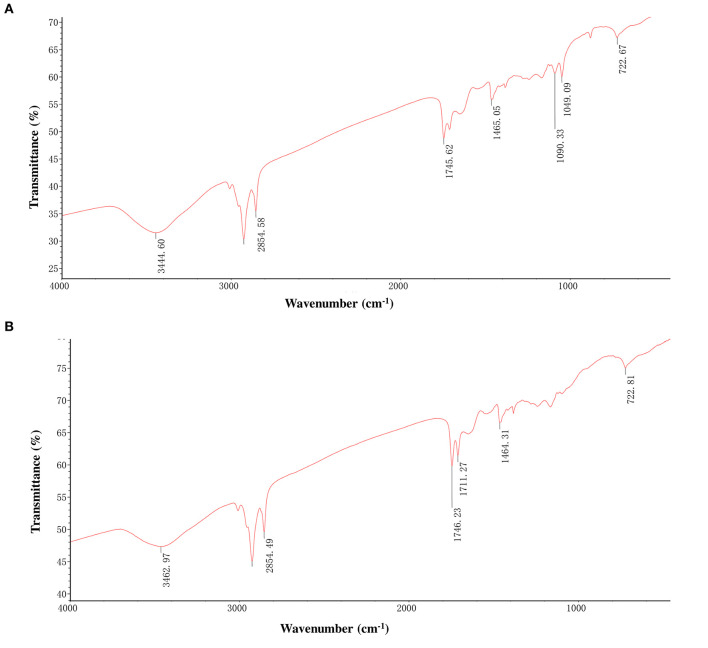
Fourier transforms infrared (FT-IR) spectra of melanins isolated from black sesame **(A)** and brown sesame **(B)** seeds.

We performed the Fourier transform infrared spectroscopy (FT-IR) test to confirm that the isolated pigment from black and brown sesame seeds is melanin. The FT-IR spectra of the isolated black and brown sesame seed's pigment were similar and are shown in Figures 3A,B. The peak at 3444.6 and 3462.97 cm^−1^ for the black and brown seeds melanin, respectively, corresponds to the OH group, and the peaks at ~2,855 cm^−1^ can be attributed to the stretching vibration of the aliphatic CH groups (14, 24, 26). The characteristic strong bands at ~1,711–1,745 cm^−1^ can be assigned to C = O stretching (26). The absorption at 1,650–1,500 cm^−1^ corresponds to the vibration of aromatic ring C = C bonds or stretching of the COOH and C-N groups, or the N-H deformation (24, 26, 32). The peaks at ~1,465 cm^−1^ are attributed to aliphatic C-H groups in the melanin pigment, and those at ~1,090 cm^−1^ are assigned to the COH stretching of phenolic compounds or aromatic rings in substituted macromolecular systems (24, 26, 33). The peak centered at 1,049 cm^−1^ for the pigment extracted from the black sesame seeds indicates CH in-plane of aliphatic structure, which is also a characteristic of melanin pigment (24). The observed peaks at ~722 cm^−1^ can be attributed to out-of-plane bending of the aromatic C-H group (24).

**Figure 4:**
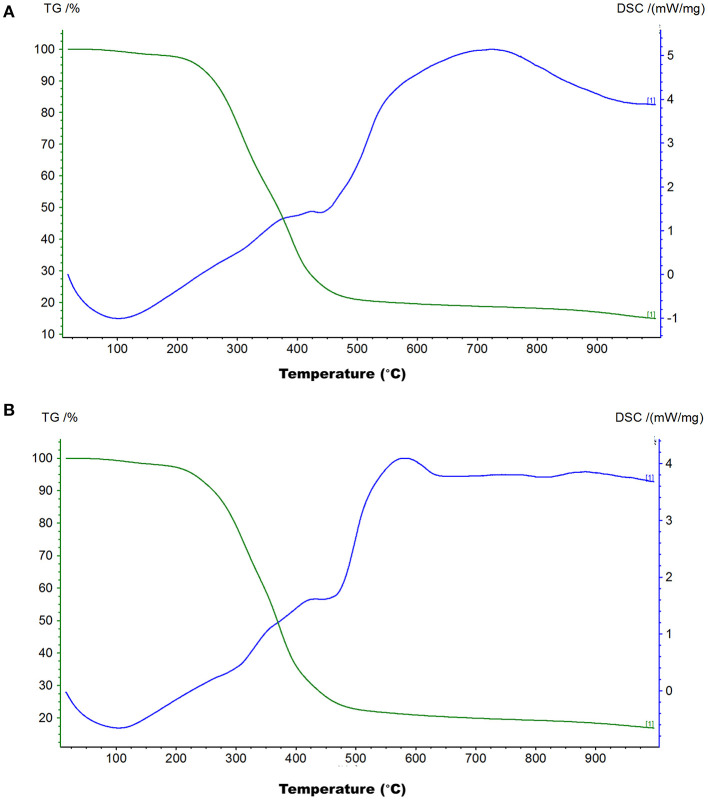
Thermogravimetry and differential scanning calorimetry (TG-DSC) curves of melanins isolated from black sesame **(A)** and brown sesame **(B)** seeds.

The TG-DSC analysis helps investigate processes that occur in a material under different temperature conditions. To check the thermal stability of the black and brown sesame seed's melanin, we carried out the TG-DSC analysis in an open system under nitrogen flow in the temperature ranges from room temperature to 1,000°C at 10°C/min heating rate. The TG and DSC curves of the black and brown seed melanin are presented in Figures 4A,B, respectively. The TG and DSC curves of the two isolated melanins showed similar trends with three sharps weight loss, one endothermal peak at ~110°C, and one broad exothermal peak. The combustion (weight loss) of the two melanins started at ~50°C. The weight decay was slight between ~50–210°C; logarithmic between ~210–410°C; and slight from ~410°C. The total weight loss of the black and brown seed's melanin was 83.12 and 85.09%, respectively, indicating the sesame melanin is heat-stable. The broad exothermal peak ranged from ~450–1,000°C.

### Comparative Metabolite Profiles of Methanol and NaOH Extracts of Sesame Seeds

Based on the results of our recent study (10) and the fact that melanin components are primarily soluble in alkaline solvents, we performed a comparative metabolomics profile analysis of methanol and NaOH extracts of black and white sesame seeds. The aim was to investigate the effect of extraction solvents on sesame seed's metabolome profile and reveal the potential precursors of the sesame melanin. The UPLC-MS/MS-based widely targeted metabolomics analysis approach (9, 31) was applied, and three varieties of black and white sesame seeds were tested (Supplementary Table S1 and Supplementary Figure S1). In general, the total number of identified metabolites varied from 351 to 403 and 462 to 468 in NaOH and 70 % methanol extracts, respectively (Supplementary Table S1). Different classes of primary and secondary metabolites were identified, among which organic and phenolic acids, lipids, amino acids and derivatives, nucleotides and derivatives, and saccharides and alcohols were predominant (Supplementary Table S2).

**Figure 5:**
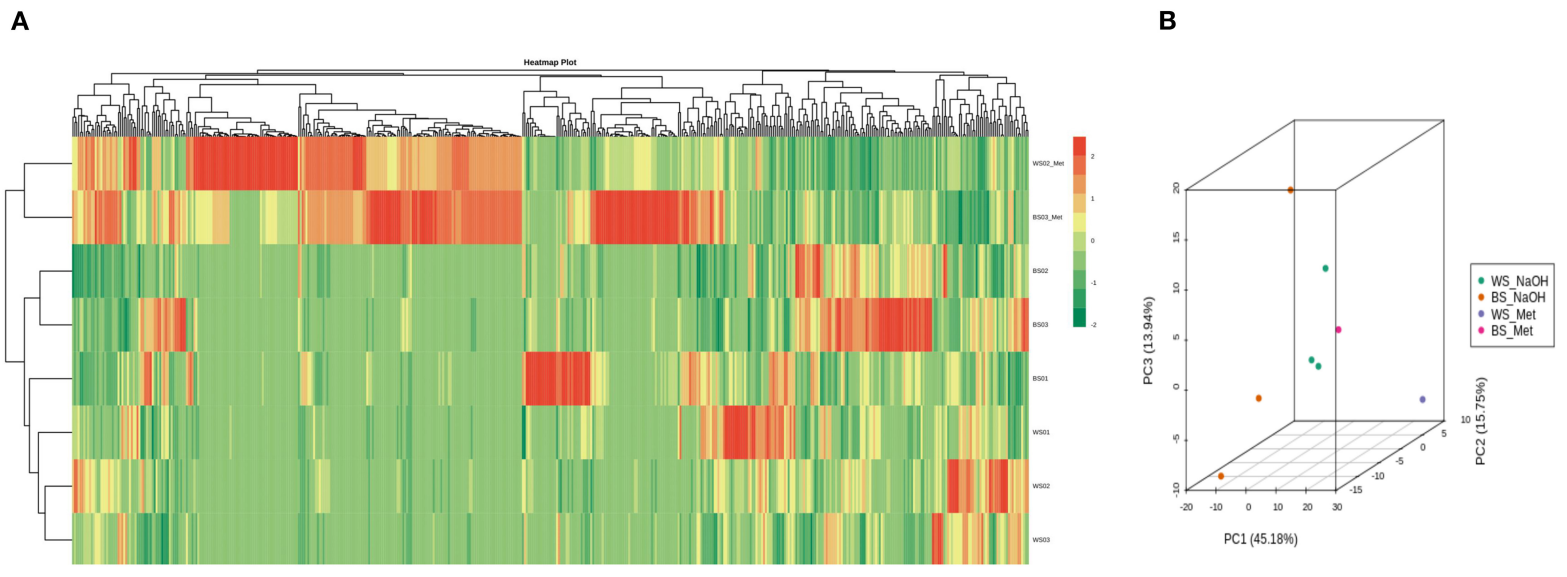
Overview of metabolites profile of sesame seeds in methanol and NaOH extracts. **(A)** Heat map visualization. The profiles of all types of metabolites were normalized to complete the hierarchical clustering. Each sample is represented by one row, and each metabolite is visualized in one column. Red indicates high abundance; green indicates relatively low metabolite abundance. **(B)** Principal component analysis (PCA) result showing metabolite profile differences between and within groups. The sample labels are defined in Supplementary Table S1.

To compare the seed's metabolites patterns in NaOH and methanol extracts, we performed the hierarchical cluster analysis (HCA) (Figure 5A). The samples extracted with 70% methanol clustered separately from those extracted with the NaOH solvent, indicating that the metabolite profiles of sesame seed in organic and aqueous solvents are different. Some metabolites exhibited high relative content in NaOH solvent. Principal component analysis (PCA) was further conducted to better understand the general metabolite variability between groups. As per the HCA result, samples extracted with NaOH and 70 % methanol were obviously separated, confirming that the metabolome profile of sesame seeds in methanol and NaOH solvents differed (Figure 5B). The HCA and PCA results also showed that metabolites accumulation in sesame seed is influenced by the genotypes.

**Figure 6:**
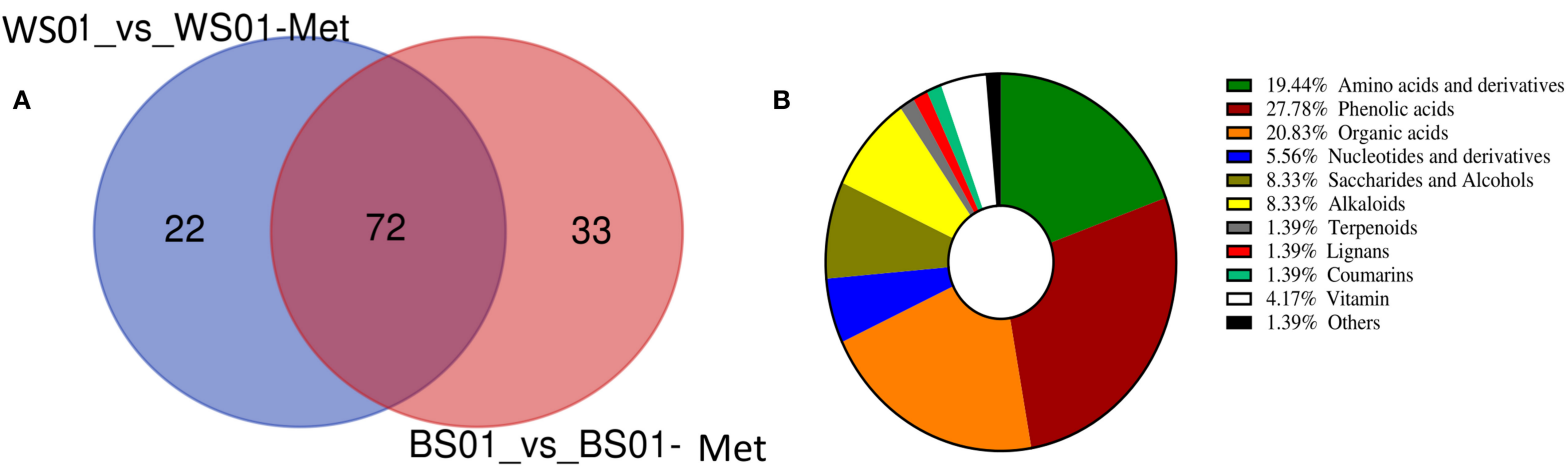
Differential metabolites between NaOH and 70 % methanol extracts of sesame seeds. **(A)** Venn diagram exhibiting the number of the key metabolites that exhibited high relative content in NaOH extract; **(B)** Classification of the 72 key metabolites that were more soluble in alkaline solution. The definition of labels is presented in Supplementary Table S1.

The differential metabolite screening was performed in the pairwise comparison between the two solvent extracts of the sesame varieties Zhongzhi28 (WS01, white seed) and Zhongzhi33 (BS01, black seed) using the criteria of fold-change (FC ≥ 2 or ≤ 0.5) and VIP > 1. The results indicated that there were 337 (94 up-regulated and 243 down-regulated) and 371 (105 up-regulated and 266 down-regulated) significantly differential metabolites between WS01-Met and WS01, and between BS01-Met and BS01, respectively. In order to identify key metabolites that were more soluble in the NaOH solvent, we constructed a Venn diagram among the up-regulated differential metabolites between WS01-Met and WS01 and between BS01-Met and BS01 (Figure 6A). There were seventy-two (72) overlapping significantly differential metabolites among the two pairwise comparisons. The overlapping metabolites are listed in Supplementary Table S3. Phenolic acids, amino acids and derivatives, and organic acids accounted for 27.78, 19.44, and 20.83%, respectively, of the key sesame seed metabolites that exhibited high relative content in the NaOH solvent extract (Figure 6B).

### Potential Precursors of the Sesame Melanin

**Figure 7:**
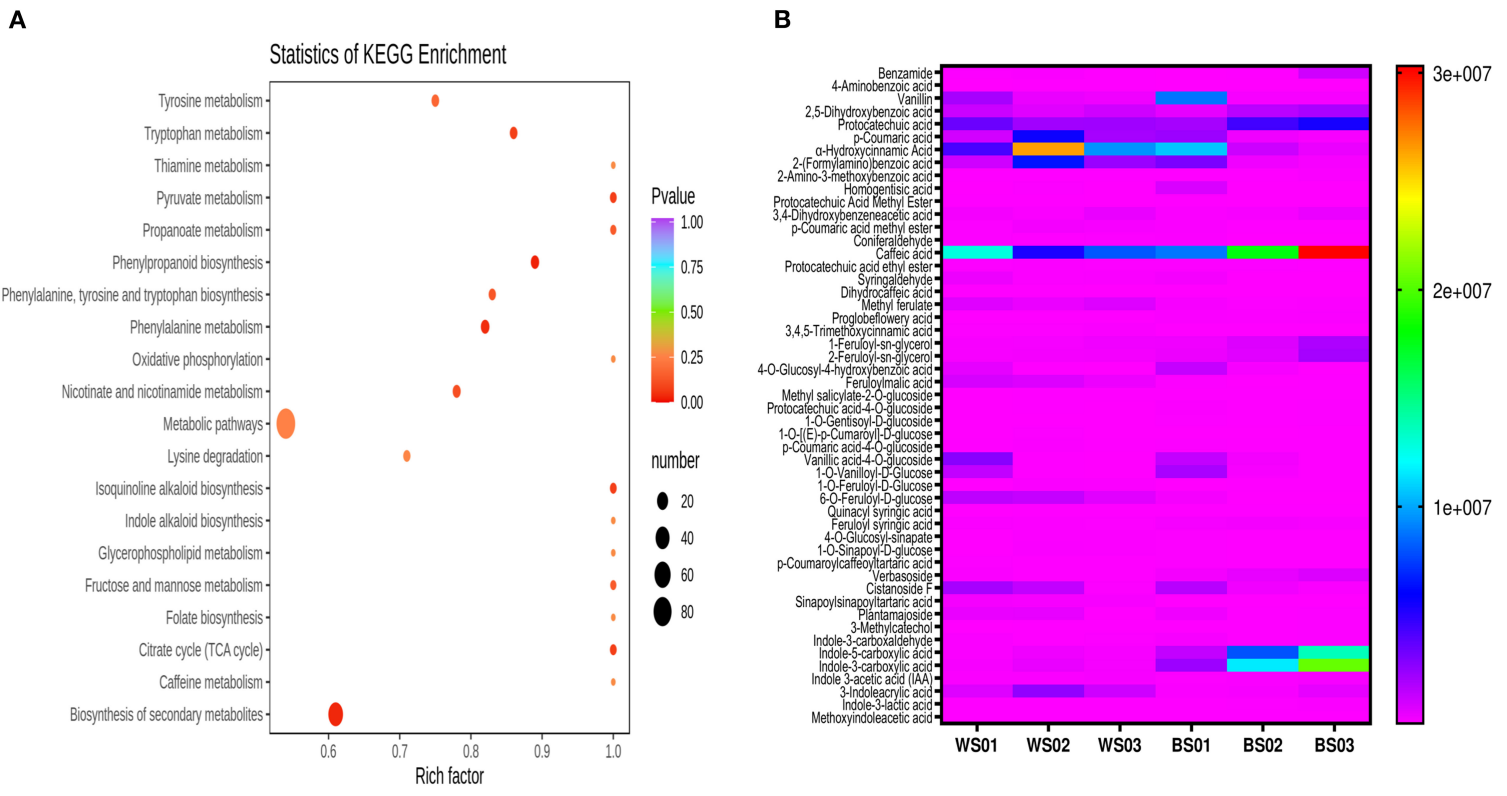
Differential accumulated metabolites (DAMs) between white and black sesame seed in NaOH extracts. **(A)** KEGG annotations and enrichment results of the DAMs between white and black sesame seeds. **(B)** Comparison of the relative content of differentially accumulated phenolic acids between white (WS) and black sesame (BS) seeds. The definition of labels is presented in Supplementary Table S1.

As melanin is exclusively soluble in alkaline solutions (24), we firstly identified the differential metabolites between the NaOH solvent extract of Zhongzhi28 (WS01, white seed) and Zhongzhi33 (BS01, black seed). Differential metabolite analysis revealed that there were 204 significant differential metabolites, including 101 up-regulated and 103 down-regulated between the two sesame (Supplementary Table S4). The KEGG pathway enrichment analysis of the differential metabolites indicated that they were mostly involved in the biosynthesis of secondary metabolites; tyrosine, tryptophan, pyruvate, and phenylalanine metabolisms; phenylpropanoid biosynthesis; and isoquinoline alkaloid biosynthesis (Figure 7A), supporting the melanin nature of the dark pigment. Considering the polyphenolic structure of melanin, we then compared the relative content of the differential phenolic acids in white (WS) and black (BS) sesame seeds (Figure 7B). The result indicated that caffeic acid, indole-3-carboxylic acid, indole-5-carboxylic acid, protocatechuic acid, homogentisic acid, benzamide, 2,5-dihydroxybenzoic acid, 3,4-dihydroxybenzeneacetic acid, and ferulic and vanillic acid derivatives accumulated highly in black sesame seeds. Therefore, we speculated that they might be the precursors of melanin in sesame.

## Discussion

Sesame is a prominent oilseed crop grown worldwide for its nutritional and therapeutical values (34, 35). Due to the huge repertory of pharmacological proprieties associated with its lignans (36–38), sesame seeds occupy an important place in food pharmacy (8). Unfortunately, the sesame seed's biological abilities are greatly influenced by the seed coat color (15, 39–41), and little is known about the dark sesame seed coat pigmentation. Therefore, this study took advantage of previous studies in sesame and other crops to isolate and characterize the dark sesame seed's pigment.

Previous studies identified PPO as the major causative gene associated with the sesame seed coat color variation (16, 18). This gene is reported to play a critical role in melanins biosynthesis in plants, including black oat, watermelon, persimmon, and black garlic (14, 24–26). In these plants, melanin was identified as the dark pigment. Therefore, we hypothesized that the dark pigment in the sesame seed coat might be melanin. Then, we successfully isolated melanin from the black and brown sesame seeds. The melanin content was higher in black sesame seeds than in brown seeds. The SEM micrographs indicated that the two melanins exhibit amorphous form without self-organization similar to those presented by Costa et al. (33). The isolated melanins were completely soluble in alkaline solutions but insoluble in water and organic solvents. Moreover, they were precipitated in FeCl_3_ solution and HCl. The solubility and precipitation results are consistent with those reported previously (20, 21, 23, 24). The black and brown sesame seed melanin's absorption spectra were similar to other plants' melanin UV-Vis spectrometry spectra, with an increase of the absorbance toward the decrease of the wavelength from 900 to 200 nm (13). FT-IR analysis demonstrated that the black and brown sesame seed's melanin displays characteristic absorbance peaks of natural melanin (24, 26, 42). In addition, thermal stability analysis confirmed the polyphenols' nature of the extracted pigments with a broad exothermic band at high-temperature (29). The TG-DSC results of the sesame melanin's were as per those of natural melanin from diverse sources (30). These findings collectively indicate that the black and brown colors of sesame seeds are mainly attributable to melanin pigments. The comparison of the metabolome profile of white, yellow, brown, and black sesame seeds revealed that the flavonoids content in sesame seed increase with the seed coat darkness, suggesting that some flavonoids might act as co-pigments to influence sesame seed coat color (10). In general, immersion of black sesame seeds in water for several hours usually induces a change of the solution color into slightly black, supporting that other compounds might contribute to the seed coat color variation in sesame. Therefore, further studies are required to clarify whether some flavonoids or other polyphenols are also involved in dark sesame seeds pigmentation.

Melanins are heterogenic polymers produced by oxidative polymerization of phenolic and indoles derived from aromatic amino acids (AAA) tyrosine, phenylalanine, and tryptophan (20, 21, 43). In this study, KEGG enrichment analysis of the deferential metabolites between black and white sesame seeds revealed that they were primarily involved in AAA metabolism, phenylpropanoid biosynthesis, and biosynthesis of secondary metabolites. These results demonstrate that AAA might play a critical role in metabolites variation and seed coat development in sesame. Moreover, they suggest that the shikimate pathway might be differently regulated during the different colored sesame seed development. Furthermore, by comparing the relative content of differential accumulated phenolics, we identified caffeic acid, indole-3-carboxylic acid, indole-5-carboxylic acid, protocatechuic acid, homogentisic acid, ferulic acid, vanillic acid, and benzoic acid as the potential precursors of melanin in sesame. 1, 4-dihydroxybenzene, catechol, and catechuic acid have been detected as the principal component of the black sesame melanin (19). Plant's melanin potential precursors include catechol, ferulic acid, gallic acid, caffeic acid, protocatechuic acid, homogentisic acid, and epigallocatechin gallate (13, 14, 24, 26).

During metabolomics analysis processes, the extraction step represents the most critical and heavily influences the results and final conclusions (6). In metabolomics, the principal idea is that samples to be analyzed comprise all the intact metabolites that can be extracted using different solvents or techniques (6). Different solvents generally lead to differences in the metabolome profile (6, 7). In this study, we investigated the metabolite profile of sesame seed in NaOH and methanol extracts. The seed's metabolite profiles were quite different in the two solvent extracts. Some metabolites showed high relative content in NaOH extract. The total number of identified metabolites varied from 462 to 468 and 351 to 403 in the methanol and NaOH extract, respectively. Differential metabolite analysis revealed that there were more than 330 significantly differential metabolites between the two solvent extracts. Some phenolic acids were mostly identified in the NaOH extract, suggesting that alkaline solution might be efficient in detecting metabolites related to the sesame seed coat. Taken together, these results suggest that a mixture of “methanol, water, and NaOH” might be the suitable solvent for metabolomics analysis of sesame seed. Further experiments are needed to validate this statement. Besides consistency with the observations in our recent study (10), we denoted that metabolites variability in sesame seed is greatly attributed to the seed coat colors and the genotypes.

## Conclusions

Overall, this study demonstrated that the main pigments responsible for seed coat color variation in sesame are melanins. Melanins were isolated from black and brown sesame seeds in a proportion of approximatively 100 and 60 mg/g, respectively. Both the melanins showed the same physicochemical proprieties as per natural melanin in other sources. They are particularly heat-stable and comprise functional groups such as N-H, O-H, C-H, COOH, and C-N. Caffeic acid, indole-3-carboxylic acid, indole-5-carboxylic acid, protocatechuic acid, homogentisic acid, ferulic acid, vanillic acid, and benzoic acid are likely the precursors of melanin in sesame. Black sesame seeds are then promising sources of edible melanin for food and biotechnological applications.

## Data Availability Statement

The original contributions presented in the study are included in the article/Supplementary Material, further inquiries can be directed to the corresponding author/s.

## Author Contributions

LW and JY: conceptualization. SD and ZL: methodology and data curation. YZ, RZ, and DL: validation and resources. ZW, AL, and WZ: formal analysis. SD: writing—original draft preparation. LW, KD, and SD: writing—review and editing. LW: project administration and funding acquisition. All authors have read and agreed to the published version of the manuscript.

## Funding

This research was funded by the Agricultural Science and Technology Innovation Project of the Chinese Academy of Agricultural Sciences (CAAS-ASTIP-2016-OCRI), the Key Research Projects of Hubei Province (2020BBA045, 2020BHB028), the Science and Technology Innovation Project of Hubei Province (201620000001048), the Fundamental Research Funds for Central Non-profit Scientific Institution (Y2019XK15-02), and the Open Project of Key Laboratory of Biology and Genetic Improvement of Oil Crops, Ministry of Agriculture and Rural Affairs, P.R. China (KF2020004).

## Conflict of Interest

The authors declare that the research was conducted in the absence of any commercial or financial relationships that could be construed as a potential conflict of interest.

## Publisher's Note

All claims expressed in this article are solely those of the authors and do not necessarily represent those of their affiliated organizations, or those of the publisher, the editors and the reviewers. Any product that may be evaluated in this article, or claim that may be made by its manufacturer, is not guaranteed or endorsed by the publisher.
