# Supplementary material for: The Dark Pigment in the Sesame (Sesamum indicum L.) Seed Coat: Isolation, Characterization, and Its Potential Precursors
Source: Front Nutr. 2022 Feb 28;9:858673. doi: 10.3389/fnut.2022.858673 (PMC8919073; doi:10.3389/fnut.2022.858673)
Supplement: Supplementary file 1 [file Presentation_1.PPTX]

## Slide 1
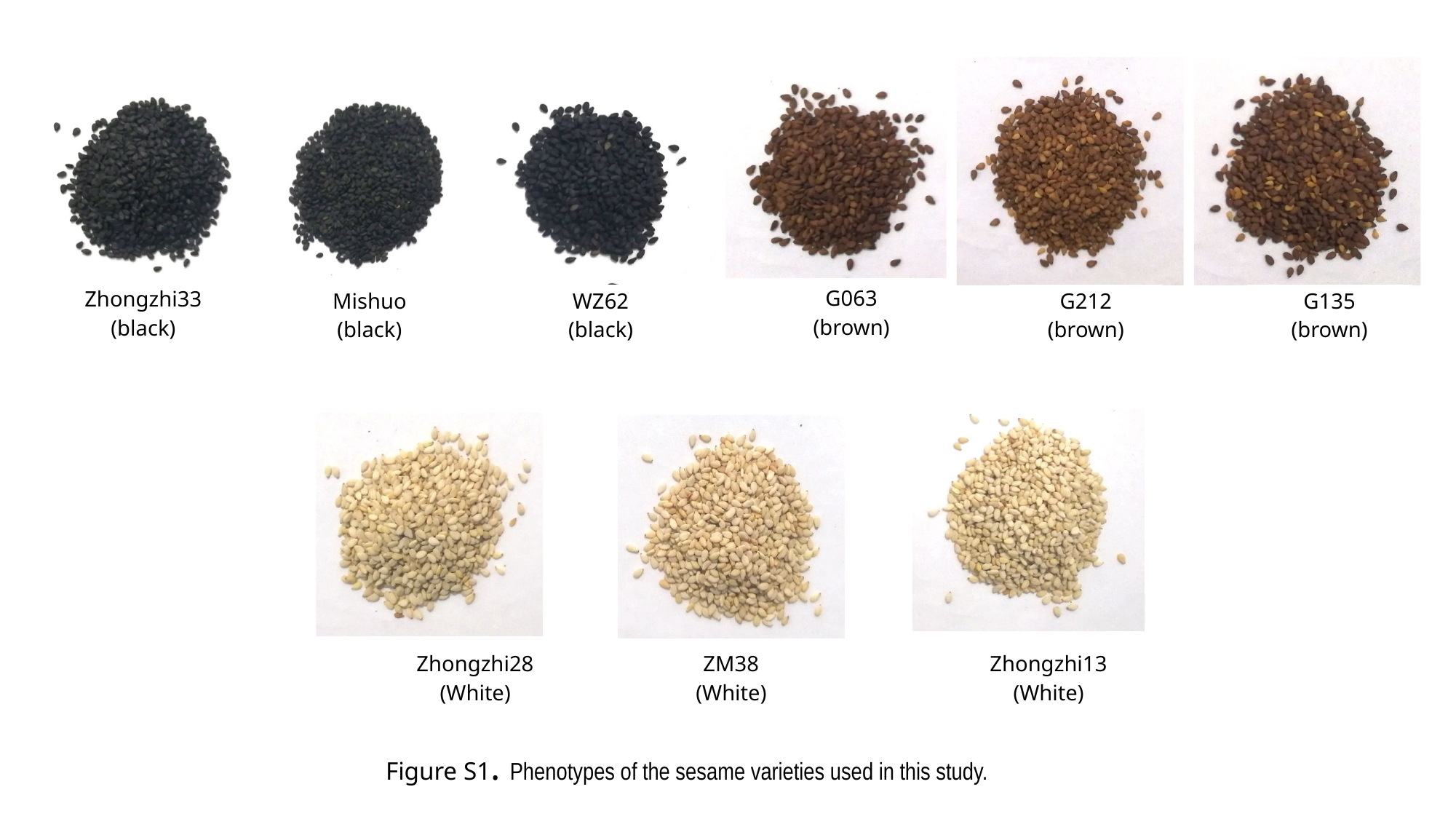

| G063 (brown) |
| --- |
| Zhongzhi33 (black) |
| --- |
| Mishuo (black) |
| --- |
| WZ62 (black) |
| --- |
| G212 (brown) |
| --- |
| G135 (brown) |
| --- |
| ZM38 (White) |
| --- |
| Zhongzhi13 (White) |
| --- |
| Zhongzhi28 (White) |
| --- |
Figure S1. Phenotypes of the sesame varieties used in this study.
